# Frontal lobe epilepsy: an eye tracking study of memory and attention

**DOI:** 10.3389/fnins.2023.1298468

**Published:** 2023-12-05

**Authors:** Qiong Zhang, Weifeng Sun, Kailing Huang, Li Qin, Shirui Wen, Xiaoyan Long, Quan Wang, Li Feng

**Affiliations:** ^1^Department of Neurology, Xiangya Hospital, Central South University, Changsha, China; ^2^National Clinical Research Center for Geriatric Disorders, Xiangya Hospital, Central South University, Changsha, Hunan, China; ^3^Key Laboratory of Biomedical Spectroscopy of Xi’an, Xi’an Institute of Optics and Precision Mechanics of the Chinese Academy of Sciences, Xi’an, China; ^4^Key Laboratory of Spectral Imaging Technology, Xi’an Institute of Optics and Precision Mechanics, Chinese Academy of Sciences, Xi’an, China; ^5^University of Chinese Academy of Sciences, Beijing, China

**Keywords:** eye tracking, memory deficit, frontal lobe epilepsy, visual attention, eye movement

## Abstract

**Objective:**

To explore the characteristics and mechanisms of working memory impairment in patients with frontal lobe epilepsy (FLE) through a memory game paradigm combined with eye tracking technology.

**Method:**

We included 44 patients with FLE and 50 healthy controls (HC). All participants completed a series of neuropsychological scale assessments and a short-term memory game on an automated computer-based memory evaluation platform with an eye tracker.

**Results:**

Memory scale scores of FLE patients including digit span (U = 747.50, *p* = 0.007), visual recognition (U = 766.50, *p* = 0.010), and logical memory (U = 544.00, *p* < 0.001) were significantly lower than HC. The patients with FLE took longer to complete the four levels of difficulty of the short-term memory game than healthy controls (level 1: U = 2974.50, *p* = 0.000; level 2: U = 3060.50, *p* = 0.000; level 3: U = 2465.00, *p* = 0.000; level 4: U = 2199.00, *p* = 0.000). During the memory decoding period, first fixation on the targets took significantly longer for FLE patients for all difficulty levels compared to controls (level 1: U = 3407.00, *p* = 0.008; level 2: U = 3618.00, *p* = 0.036; level 3: U = 3345.00, *p* = 0.006; level 4: U = 2781.00, *p* = 0.000). The average fixation duration per target among patients with FLE was found to be significantly longer compared to HC (level 1: U = 2994.50, *p* = 0.000; level 2: U = 3101.00, *p* = 0.000; level 3: U = 2559.50, p = 0.000; level 4: U = 2184.50, *p* = 0.000). The total fixation duration on AOI/total completion time of FLE patients was significantly lower than those of HC for levels 1 to 3 (level 1: U = 1557.00, *p* = 0.000; level 2: U = 2333.00, *p* = 0.000; level 3: U = 2757.00, *p* = 0.000). Furthermore, the eye tracking data during the memory decoding phase were correlated with neuropsychological scale scores (*p* < 0.05).

**Conclusion:**

Patients with FLE exhibited short-term memory impairment probably due to deficits in attentional maintenance, especially during the memory decoding phase. Eye tracking technology provided the possibility to help separate and quantify visual attention from memory processing, contributing to exploring underlying mechanisms of memory impairment in FLE.

## 1 Introduction

Frontal lobe epilepsy (FLE) is the second most prevalent focal epilepsy in adults, accounting for approximately 20–30% of cases (Giovagnoli et al., 2020; McGonigal, 2022). It is increasingly recognized as a brain network disorder, which impacts a broad range of cognitive domains (Rayner et al., 2015; Nair and Szaflarski, 2020). Although attention deficits and impaired executive functioning are relatively common, memory deficits have also been commonly described among patients with FLE (Kibby et al., 2019). However, the frontal lobe is not traditionally the brain area responsible for memory function (Buschman and Miller, 2007; Centeno et al., 2010; Al-Aidroos et al., 2012; Fiebelkorn and Kastner, 2020). Therefore, how memory can be impaired in FLE and whether patients have specific memory characteristics remain inconclusive.

Previous studies have demonstrated that attention plays a crucial role in the process of memory formation (Zelinsky and Loschky, 2005; Chun and Johnson, 2011; Bahmani et al., 2019). More specifically, visual attention fundamentally determines which information is inputted and encoded into memory (Hollingworth et al., 2001; Voss et al., 2017). Rock and Gutman (1981) have proven that people can better recall items that have received more visual attention. Bahmani et al. (2019) also confirmed the inextricable relationship between memory and visual attention at the electrophysiological, behavioral, and anatomical levels. However, the conventional scales of memory evaluations, which include the Wechsler Memory Scale (WMS), the Hopkins Verbal Learning Test, and the Rey Auditory Verbal Learning Test, are unable to separate visual attention from memory and lack the ability to gauge and accurately measure it (Sherman et al., 2012; McAuley et al., 2015; Sveikata et al., 2019). Consequently, a tool that can quantitatively measure visual attention during memory processes and assist us in investigating the influence of attention deficit on memory impairment is urgently needed.

Eye tracking technology has the ability to capture visual behaviors and track the path of an individual’s eye movements in real time as patients engage in cognitive tasks (Boraston and Blakemore, 2007; Oyama et al., 2019; Tao et al., 2020). When integrated with memory tasks, it can accurately and comprehensively quantify visual attention (Boraston and Blakemore, 2007; Blais et al., 2008). Sylvia B. Guillory applied eye tracking-based measurement in the study of memory function among individuals with Autism Spectrum Disorder (ASD), specifically those with Phelan-McDermid Syndrome (PMS) (Guillory et al., 2021). They revealed that patients with PMS and co-morbid ASD diagnoses have poorer memory and lower attentional engagement with social images (Guillory et al., 2021). Using oculomotor tracking combined with a visual paired-comparison paradigm, patients with Rett syndrome showed restricted and immature visual attention characteristics during recognition memory tasks (Rose et al., 2013). In our previous work on an automated computer-based memory assessment platform with an eye tracker, we found that TLE patients experienced memory retrieval difficulties with relative sparing of attention (Zhu et al., 2021; Xiao et al., 2023).

In the present study, we hypothesize that patients with FLE might have a specific memory impairment profile, which is different from those of TLE, and attention might contribute to deficits in the memory process in FLE. To this end, we first applied the digit span, visual recognition, and logical memory scales, as per the Wechsler Memory Scales-Chinese Revision (WMS-RC), in patients with FLE. To further delineate the patterns of memory impairment, we employed an automated memory assessment platform, which, relying on eye tracking technology, recorded and analyzed associated parameters in both memory encoding and decoding phases, including total completion time, first fixation time on target, average fixation duration per target, etc. We made a correlation analysis between the eye tracking index and the performance of memory scales. Our work showed that patients with FLE exhibited impaired short-term memory function, which might be mostly due to deficits in attention maintenance, especially during the memory decoding phase.

## 2 Methods

### 2.1 Participants

A total of 44 FLE patients [22 male, age 28.11 (9.15)] and 50 healthy controls (HC) [22 male, age 30.22 (12.39)] were enrolled in our study and were matched for age, sex (male/female), and years of education. All FLE patients were recruited from September 2020 to August 2021 at Xiangya Hospital, Central South University, and were diagnosed with FLE according to the definition of the International League Against Epilepsy (ILAE) by two trained epileptologists (Fisher et al., 2017). The diagnostic criteria for FLE were based on history (typical symptoms of seizures suggestive of frontal lobe origin), EEG, and neuroimaging. Exclusion criteria: (1) those aged under 16 years or over 60 years; (2) those who had a history of neurological disorders other than epilepsy and a history of neurosurgery; (3) those who claimed to have subjective memory impairment; (4) those who also had psychiatric disorders or comorbidities; (5) those who failed the eye tracking calibration procedure or were unable to understand the experimental procedure.

### 2.2 Memory scale assessment

All the participants undertook a memory scale assessment. We applied digit span, visual recognition, and logical memory scales, as per the Wechsler Memory Scales-Chinese Revision (WMS-RC), to assess the memory ability of the participants.

Digit span aims to measure verbal working memory and can be conducted forward and backward. In digit span forward, the participants are required to repeat a string of digits in sequence after hearing them, starting by repeating 3 digits, with a maximum of 11 digits, and there are two chances for each digit length. In digit span backward, the participants are required to repeat the digits in reverse, starting with 2 digits, with a maximum of 10. The test was terminated when 11 digits were correctly recalled or incorrectly recalled twice in a row.

Visual recognition measures visuospatial memory. In this task, participants were shown eight cards for 30 s and were required to recall the eight cards in 28 cards. The cards included graphics, Chinese characters, and mathematical symbols. The more cards the participant recognized, the higher the score.

Logical memory task measured verbal episodic memory. The participants were asked to browse a short story, and the investigator would read it aloud at the same time. When the investigator finished reading, the story card would be taken back and the participants were required to immediately recall the story in as much detail as possible. There were two different short stories for each trial. The more details the participant recalled, the higher the score.

### 2.3 Memory paradigm

An automated computer-based memory assessment platform was adapted from Li et al., as reported in our previous study, for measuring short-term memory. The stimuli included 38 front-facing human images downloaded from the Chinese University of Hong Kong (CUHK) student database^1^ and 38 images of fractals downloaded from the Web.

**Figure 1:**
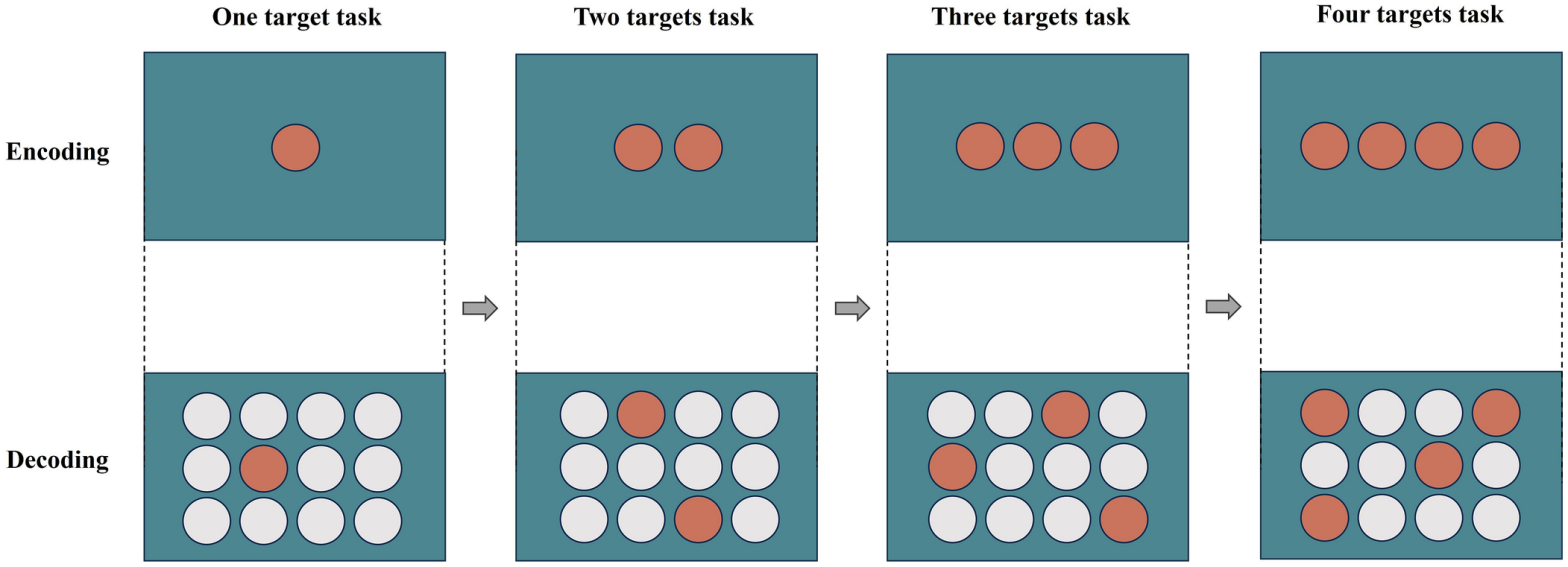
During the trial, a target was shown on the screen for 5 s (encoding), followed by the appearance of 12 (4 × 3) potential answers. Participants were required to recall and click on the correct target from the 12 objects (decoding). The targets were numbered from 1 to 4, and participants were required to choose all the correct targets during the decoding phase in order to proceed to the next level. The orange circle indicates the target and the white circle in the decoding phase is the interference graphic.

The participants were presented with the image(s) for 5 s (referred to as the encoding phase); then, the image(s) would disappear and 12 images in 3 rows and 4 columns would be presented on the screen (referred to as the decoding phase). They were required to choose the image they memorized by clicking the mouse, they could not proceed with the next trial until all the correct targets had been clicked. There are four difficulty levels in the short-term memory game, starting with memorizing one target (level 1) and ending with memorizing four memory targets (level 4), with four trials in each level (Figure 1). The terms “YES” and “NO” are presented as feedback for the correct selections and incorrect selections, respectively.

### 2.4 Eye tracking task

#### 2.4.1 Eye tracking recording

Concurrently with the assessment of short-term memory, we gathered real-time eye tracking information utilizing the EyeLink 1,000 Plus eye tracker to observe the visual search conducted by the participants throughout the task. The eye tracking data was captured in monocular remote mode with a sampling rate of 500 Hz. To ensure accuracy, participants were instructed to minimize movement of their upper body while using the remote mode. Minor movements of the head and upper body were permitted, as the oculomotor system can be automatically calibrated. A standard 9-point calibration procedure was implemented to guarantee the precision and accuracy of the collected data. Participants who were unable to pass the calibration process were excluded from the subsequent experiment.

#### 2.4.2 Eye tracking analysis

Eye tracking data were analyzed with DataViewer (version 4.2). We extracted the eye tracking indicators for the encoding and decoding phases separately. Fixation, saccade, and other behaviors were directly calculated using the EyeLink system. The EyeLink system uses three thresholds to detect saccades and fixation: motion, velocity, and acceleration, corresponding to 0.15 degrees, 30 degrees/s, and 8,000 degrees/s^2^, respectively. The update interval and data accumulation period of the whole system are set at 50 milliseconds. The eye tracking indicators included total completion time, first fixation time on target, average fixation duration per target, average fixation count per target, total fixation duration on area of interest (AOI)/total completion time, and average fixation duration per target/total fixation duration on AOI. First fixation time on target refers to the time point when the participants notice the presented target. The reaction time refers to the duration between the introduction of a stimulus and the subsequent response. The first fixation time on target in the encoding phase can be used to reflect the subject’s response and alertness when participating in the experiment. Average fixation duration per target is the average fixation durations within per target. Average fixation count per target is the average number of fixations that fall within the target. Total fixation duration on AOI/total completion time is the percentage of total fixation duration on AOI to total completion time. Average fixation duration per target/total fixation duration on AOI is the percentage of average fixation duration per target to the total fixation duration on AOI. We used Python (version 3.7.10) to process the eye tracking data. For each participant, the eye tracking indicators were averaged over the trials to obtain the average indicator corresponding to each difficulty level. We also extracted the above eye tracking indicators under memorized fractal images and memorized front-facing images.

### 2.5 Statistical analysis

The statistical analysis was conducted using the SPSS software package (version 27.0; SPSS Inc., Chicago, Illinois, United States). In order to analyze categorical variables such as sex in both FLE patients and healthy controls, χ2 analysis was employed. Continuous variables such as age and years of education, which passed the normality test, were analyzed using an independent samples t-test. The data were presented as mean and standard deviation (Mean ± SD). For memory scale scores and eye tracking data that significantly deviated from normal distribution, the Mann–Whitney U test was used to compare the differences between the two groups. Spearman’s correlation analysis was used to determine the correlation between the eye tracking indicators and the memory scale score. Two-tailed *p*-values were calculated for all tests, and p-values less than 0.05 were considered statistically significant.

## 3 Results

### 3.1 Participant demographics

A total of 44 adult FLE patients and 50 HC eligible for this study were included. The demographic and clinical characteristics of the participants are shown in Table 1. No statistically significant differences were found between FLE patients and HC regarding sex (*p* = 0.561), age (*p* = 0.341), and years of education (*p* = 0.079).

**Table 1:** Demographic and clinical characteristics of participants.

	FLE (*n* = 44)	HC (*n* = 50)	*p*-value
Male, *n* (%)	22 (50)	22 (44)	0.561
Age, y, mean ± SD	28.11 ± 9.15	30.22 ± 12.39	0.347
Education, y, mean ± SD	11.84 ± 2.91	13.00 ± 3.36	0.079
Age at onset, y, mean ± SD	16.21 ± 10.02	—	—
Duration, y, mean ± SD	11.93 ± 10.55	—	—
Side of epilepsy foci, *n* (%)			
Left	18 (41)	—	—
Right	16 (36)	—	—
Bilateral/Unclear	10 (23)	—	—
Number of ASMs used in treatment, n, mean ± SD	1.43 ± 1.02	—	—

FLE, frontal lobe epilepsy; HC, healthy control; ASM, anti-seizure medication; SD, standard deviation.

### 3.2 Memory scale assessment

As shown in Table 2, there were significant differences between FLE patients and HC in the scores of scales reflecting memory function, including digit span (U = 747.50, *p* = 0.007), visual recognition (U = 766.50, *p* = 0.010), and logical memory (U = 544.00, *p* < 0.001). The FLE patients’ scores in these scales were significantly lower than those of HC.

**Table 2:** Memory scale assessment.

	FLE (*n* = 44)	HC (*n* = 50)	test	*p*-value
Digit span, median (P_25_, P_75_)	13.00 (11.00, 15.75)	15.00 (13.00, 18.00)	U = 747.50	0.007^**^
Visual Recognition, median (P_25_, P_75_)	14.00 (13.00, 15.00)	15.00 (14.00, 16.00)	U = 766.50	0.010^*^
Logical Memory, median (P_25_, P_75_)	10.50 (5.75, 12.38)	13.75 (10.88, 16.00)	U = 544.00	<0.001^***^

FLE, frontal lobe epilepsy; HC, healthy control.

### 3.3 Short-term memory task

**Figure 2:**
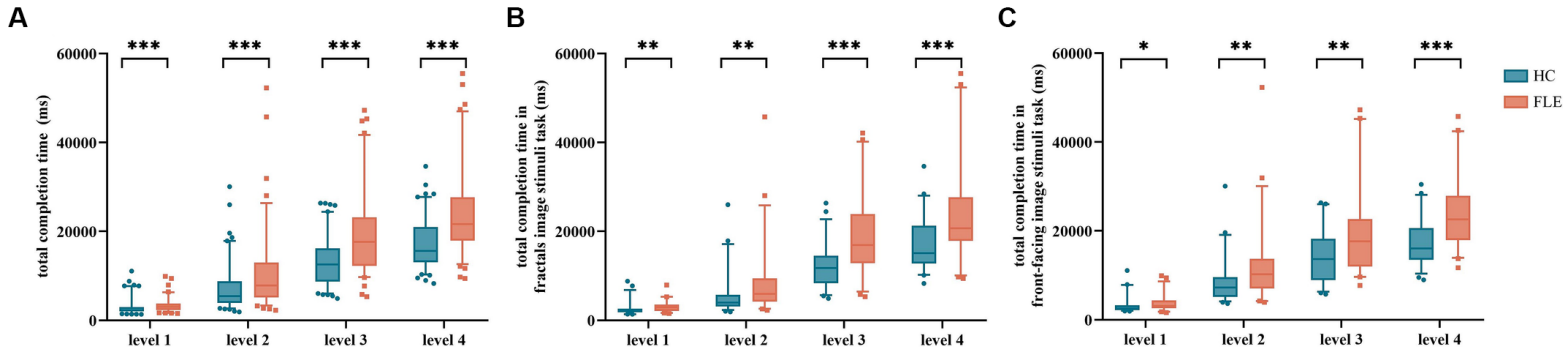
Comparison of **(A)** total completion time of the Short-Term Memory task, **(B)** total completion time under fractal image stimuli, and **(C)** total completion time under front-facing image stimuli between the HC group and FLE group at four difficulty levels. Statistical significance for each task between groups is indicated by asterisk(s) (^*^p < 0.05, ^**^p < 0.01, ^***^p < 0.001). HC, healthy control; FLE, frontal lobe epilepsy.

There was a significant difference in total completion time between the two groups in the decoding phase in the four difficulty levels (level 1: U = 2974.50, *p* = 0.000; level 2: U = 3060.50, *p* = 0.000; level 3: U = 2465.00, *p* = 0.000; level 4: U = 2199.00, *p* = 0.000). The total completion time of the FLE patients in the four difficulty levels was significantly longer than that of the HC (Figure 2A).

The total completion time of FLE patients under fractal image stimuli was significantly longer than that of HC in levels 1–4 (level 1: U = 653.00, *p* = 0.001; level 2: U = 697.00, *p* = 0.002; level 3: U = 509.00, *p* = 0.000; level 4: U = 569.00, p = 0.000) (Figure 2B). The total completion time of FLE patients under front-facing image stimuli was also significantly longer than that of HC in levels 1–4 (level 1: U = 794.00, *p* = 0.020; level 2: U = 732.00, *p* = 0.005; level 3: U = 710.00, *p* = 0.003; level 4: U = 535.00, *p* = 0.000) (Figure 2C).

### 3.4 Eye tracking memory task indicators

#### 3.4.1 Indicators of the encoding phase

In the memory encoding phase, the first fixation time on targets of FLE patients was significantly longer than that of HC in level 4 (U = 3603.5, *p* = 0.032), while no significant difference was found in the remaining difficulty levels. There were no differences in average fixation duration per target and average fixation count per target in levels 1 to 4 (all *p* > 0.05). When it comes to different image stimuli, no differences were found in all eye tracking indicators in the four difficulty levels under both fractal image stimuli and front-facing image stimuli (Table 3).

**Table 3:** Comparison of eye tracking indicators in encoding of participants.

	FLE (*n* = 44)	HC (*n* = 50)	test	*p*-value
First fixation time on target in level 1, ms	513.50 (300.28, 633.13)	566.50 (356.63, 768.13)	U = 3795.50	0.104
First fixation time on targets in level 2, ms	715.00 (500.19, 933.94)	710.75 (561.31, 915.10)	U = 4307.00	0.803
First fixation time on targets in level 3, ms	719.75 (540.92, 893.42)	670.75 (551.08, 845.21)	U = 4062.00	0.364
First fixation time on targets in level 4, ms	1013.44 (807.31, 1283.81)	922.69 (767.18, 1084.09)	U = 3603.50	0.032^*^
total fixation duration on target in level 1, ms	4351.75 (3923.38, 4551.50)	4262.50 (3835.00, 4494.88)	U = 3909.50	0.188
total fixation duration on target in level 2, ms	2117.13 (1920.69, 2243.13)	2107.63 (1952.69, 2196.19)	U = 4154.00	0.509
total fixation duration on target in level 3, ms	1665.08 (1479.58, 1790.83)	1658.25 (1490.96, 1744.42)	U = 4271.50	0.730
total fixation duration on target in level 4, ms	1338.25 (1207.31, 1407.56)	1321.69 (1229.31, 1397.00)	U = 4398.50	0.997
total number of fixations on target in level 1	11.50 (9.50, 13.44)	11.88 (9.00, 13.69)	U = 4249.00	0.685
total number of fixations on target in level 2	6.44 (5.34, 7.38)	6.48 (5.41, 7.38)	U = 4326.00	0.842
total number of fixations on target in level 3	5.36 (4.60, 6.17)	5.53 (5.00, 6.29)	U = 4123.00	0.457
total number of fixations on target in level 4	4.44 (3.78, 5.13)	4.64 (3.89, 5.25)	U = 4050.50	0.348

FLE, frontal lobe epilepsy; HC, healthy control.

#### 3.4.2 Indicators of the decoding phase

**Figure 3:**
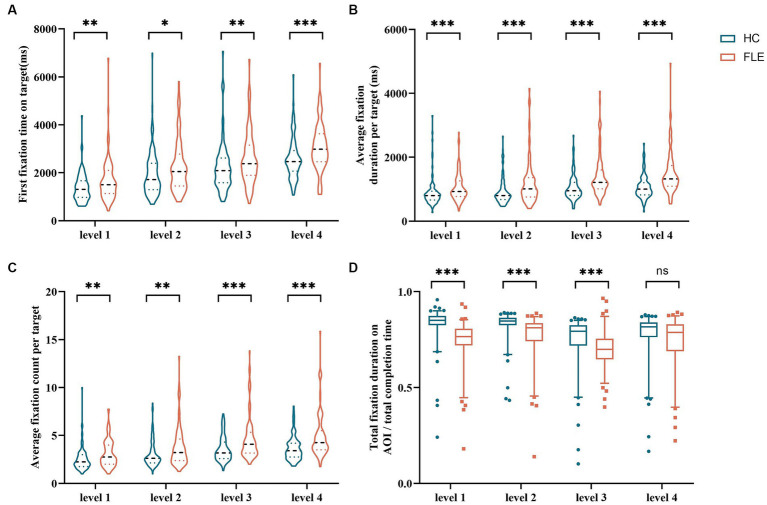
Comparison of **(A)** first fixation time on target, **(B)** average fixation duration per target, **(C)** average fixation count per target, and **(D)** total fixation duration on AOI/total completion time of the decoding phase in all tasks between the HC group and FLE group. Statistical significance for each task between groups is indicated by asterisk(s) (^*^p < 0.05, ^**^p < 0.01, ^***^p < 0.001). HC, healthy control; FLE, frontal lobe epilepsy.

The first fixation time on targets of FLE patients was significantly longer than that of HC in the four difficulty levels (level 1: U = 3407.00, *p* = 0.008; level 2: U = 3618.00, *p* = 0.036; level 3: U = 3345.00, *p* = 0.006; level 4: U = 2781.00, *p* = 0.000). The average fixation duration per target of FLE patients was significantly longer than that of HC (level 1: U = 2994.50, *p* = 0.000; level 2: U = 3101.00, *p* = 0.000; level 3: U = 2559.50, *p* = 0.000; level 4: U = 2184.50, *p* = 0.000), the average fixation count per target of FLE patients were also significantly higher than that of HC (level 1: U = 3206.50, *p* = 0.001; level 2: U = 3320.00, *p* = 0.004; level 3: U = 2855.50, *p* = 0.000; level 4: U = 2571.50, *p* = 0.000). The total fixation duration on AOI/total completion time of FLE patients was significantly lower than that of HC in levels 1 to 3 (level 1: U = 1557.00, *p* = 0.000; level 2: U = 2333.00, *p* = 0.000; level 3: U = 2757.00, *p* = 0.000), while no difference was found in level 4 (level 4: U = 3683.00, *p* = 0.054). The average fixation duration per target/total fixation duration on AOI of FLE patients was significantly higher than that of HC in level 1 (U = 3987.00, *p* = 0.020), while no differences were found in the remaining difficulty levels (Table 4 and Figure 3).

**Table 4:** Comparison of memory game and eye tracking indicators in decoding of participants.

	FLE (*n* = 44)	HC (*n* = 50)	test	*p*-value
total completion time in level 1, ms	3022.75 (2291.25, 3771.38)	2283.25 (1998.88, 3006.38)	U = 2974.50	0.000^***^
total completion time in level 2, ms	7848.75 (5157.63, 13023.63)	5475.25 (3917.88, 8806.88)	U = 3060.50	0.000^***^
total completion time in level 3, ms	18083.00 (12327.00, 24037.75)	12554.92 (8626.63, 16203.63)	U = 2465.00	0.000^***^
total completion time in level 4, ms	22887.25 (17968.38, 28236.88)	16073.25 (13467.38, 20581.25)	U = 2199.00	0.000^***^
First fixation time on target in level 1, ms	1504.33 (1135.75, 2098.50)	1311.50 (974.88, 1669.63)	U = 3407.00	0.008^**^
First fixation time on targets in level 2, ms	2048.13 (1451.13, 2781.50)	1715.00 (1296.75, 2397.44)	U = 3618.00	0.036^*^
First fixation time on targets in level 3, ms	2381.33 (1895.04, 3162.79)	2076.00 (1577.33, 2625.00)	U = 3345.00	0.000^***^
First fixation time on targets in level 4, ms	3160.35 (2632.72, 3759.44)	2655.75 (2394.00, 3218.91)	U = 2718.00	0.020^*^
Average fixation duration per target in level 1, ms	926.25 (770.25, 1262.25)	800.00 (662.00, 939.50)	U = 2994.50	0.000^***^
Average fixation duration per target in level 2, ms	1009.38 (758.31, 1362.13)	801.88 (678.25, 1013.38)	U = 2101.00	0.000^***^
Average fixation duration per target in level 3, ms	1216.33 (1018.46, 1611.68)	951.42 (804.42, 1215.79)	U = 2559.50	0.000^***^
Average fixation duration per target in level 4, ms	1348.13 (1093.53, 1739.31)	1093.81 (1358.03,892.75)	U = 2184.50	0.000^***^
Average fixation count per target in level 1	2.75 (2.00, 4.00)	2.25 (1.75, 3.00)	U = 3206.50	0.001^**^
Average fixation count per target in level 2	3.21 (2.38, 4.63)	2.63 (2.14, 3.56)	U = 3320.00	0.004^***^
Average fixation count per target in level 3	4.08 (3.17, 5.46)	3.18 (2.58, 4.31)	U = 2855.50	0.000^***^
Average fixation count per target in level 4	4.66 (3.78, 6.19)	3.66 (2.95, 4.56)	U = 2571.50	0.000^***^
Total fixation duration on AOI/ total completion time in level 1, ratio	0.77 (0.72, 0.81)	0.85 (0.83, 0.87)	U = 1557.00	0.000^***^
Total fixation duration on AOI/ total completion time in level 2, ratio	0.81 (0.74, 0.84)	0.85 (0.82, 0.86)	U = 2333.00	0.000^***^
Total fixation duration on AOI/ total completion time in level 3, ratio	0.70 (0.65, 0.75)	0.79 (0.72, 0.83)	U = 2757.00	0.000^***^
Total fixation duration on AOI/ total completion time in level 4, ratio	0.79 (0.64, 0.84)	0.82 (0.77, 0.85)	U = 3683.00	0.054
Average fixation duration per target/total fixation duration on AOI in level 1, ratio	0.46 (0.33, 0.56)	0.40 (0.32, 0.48)	U = 3987.00	0.020^*^
Average fixation duration per target/total fixation duration on AOI in level 2, ratio	0.16 (0.13, 0.22)	0.18 (0.14, 0.21)	U = 3426.00	0.413
Average fixation duration per target/total fixation duration on AOI in level 3, ratio	0.11 (0.10, 0.12)	0.11 (0.10, 0.13)	U = 2918.00	0.117
Average fixation duration per target/total fixation duration on AOI in level 4, ratio	0.09 (0.07, 0.10)	0.08 (0.07, 0.10)	U = 3279.00	0.677

FLE, frontal lobe epilepsy; HC, healthy control; AOI, area of interest.

##### 3.4.2.1 Fractal image stimuli task

**Figure 4:**
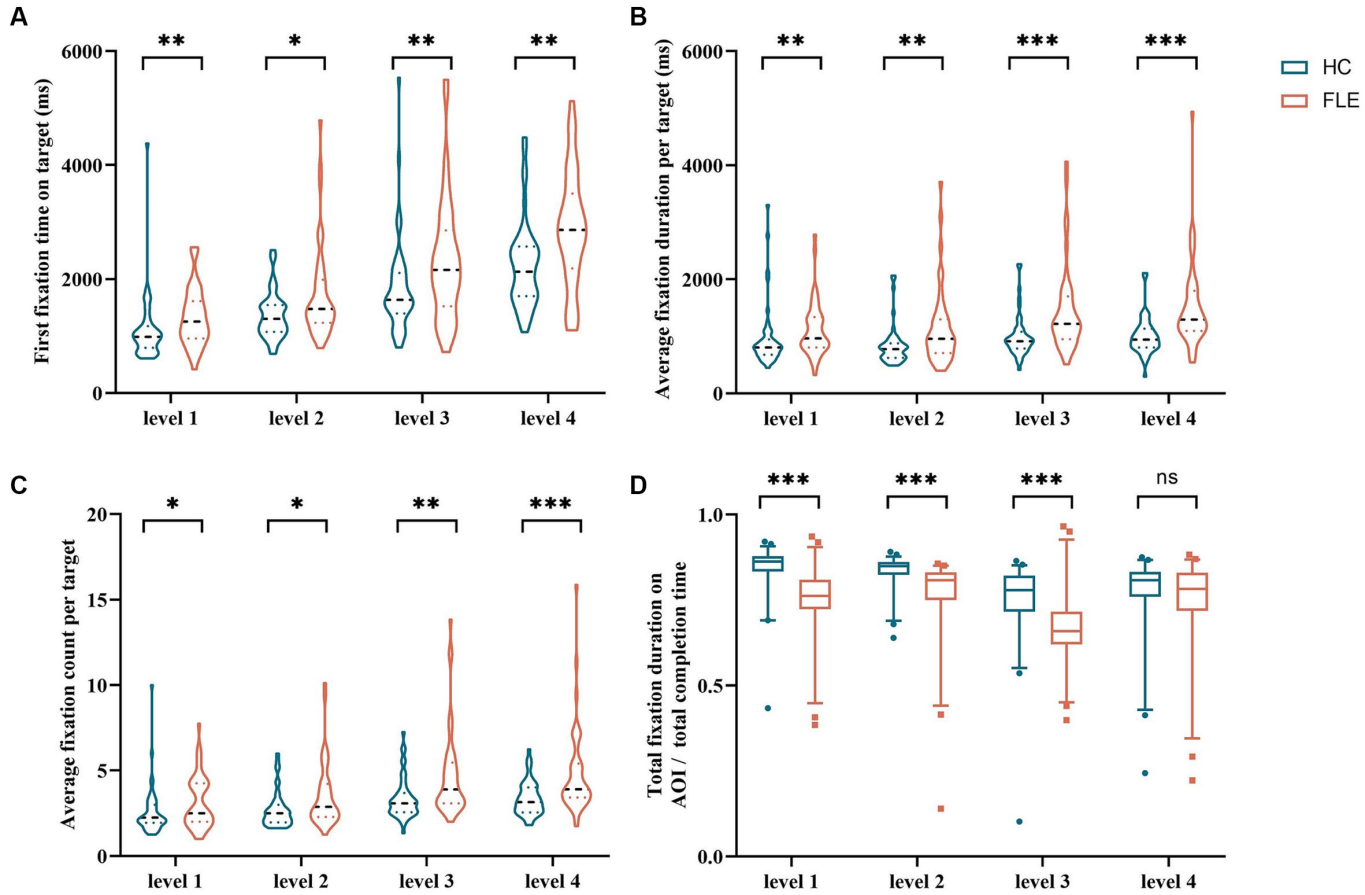
Comparison of **(A)** first fixation time on target, **(B)** average fixation duration per target, **(C)** average fixation count per target, and **(D)** total fixation duration on AOI/total completion time of the decoding phase in the fractal image stimuli task between the HC group and FLE group. Statistical significance for each task between groups is indicated by asterisk(s) (^*^p < 0.05, ^**^p < 0.01, ^***^p < 0.001). HC, healthy control; FLE, frontal lobe epilepsy.

The first fixation time on target of FLE patients was significantly longer than that of HC (level 1: U = 695.00, *p* = 0.002; level 2: U = 798.50, *p* = 0.022; level 3: U = 728.00, *p* = 0.007; level 4: U = 652.00, *p* = 0.001). The average fixation duration per target of FLE patients was significantly longer than that of HC (level 1: U = 720.00, *p* = 0.004; level 2: U = 709.00, *p* = 0.003; level 3: U = 578.00, *p* = 0.000; level 4: U = 459.00, *p* = 0.000), and the average fixation count per target of FLE patients was significantly higher than that of HC (level 1: U = 829.00, *p* = 0.039; level 2: U = 761.50, *p* = 0.010; level 3: U = 655.00, *p* = 0.001; level 4: U = 597.00, *p* = 0.000). The total fixation duration on AOI/total completion time of FLE patients was significantly lower than that of HC (level 1: U = 369.00, *p* = 0.000; level 2: U = 463.00, *p* = 0.000; level 3: U = 544.00, *p* = 0.000) **(**Figure 4**)**.

##### 3.4.2.2 Front-facing image stimuli task

**Figure 5:**
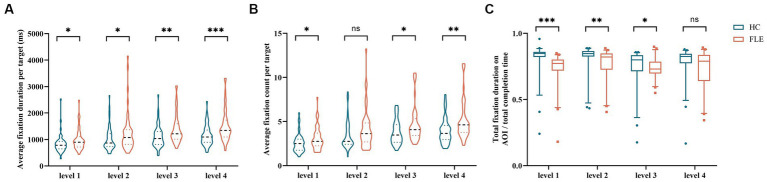
Comparison of **(A)** average fixation duration per target, **(B)** average fixation count per target, and **(C)** total fixation duration on AOI/total completion time of the decoding phase in the front-facing image stimuli task between the HC group and FLE group. Statistical significance for each task between groups is indicated by asterisk(s) (^*^p < 0.05, ^**^p < 0.01, ^***^p < 0.001). HC, healthy control; FLE, frontal lobe epilepsy.

The first fixation time on target of FLE patients was significantly longer than that of HC (level 4: U = 745.00, p = 0.010), the average fixation duration per target of FLE patients was significantly longer than that of HC (level 1: U = 774.50, *p* = 0.014; level 2: U = 836.00, *p* = 0.045; level 3: U = 723.50, p = 0.004; level 4: U = 636.50, p = 0.000), the average fixation count per target of FLE patients was significantly higher than that of HC (level 1: U = 783,50, *p* = 0.016; level 3: U = 783.00, p = 0.016, level 4: U = 636.50, p = 0.000). The total fixation duration on AOI/total completion time of FLE patients was significantly lower than that of HC (level 1: U = 395.00, p = 0.000; level 2: U = 695.00, *p* = 0.002; level 3: U = 799.00, *p* = 0.023) (Figure 5).

### 3.5 Correlation of memory scale assessment and eye tracking indicators

**Figure 6:**
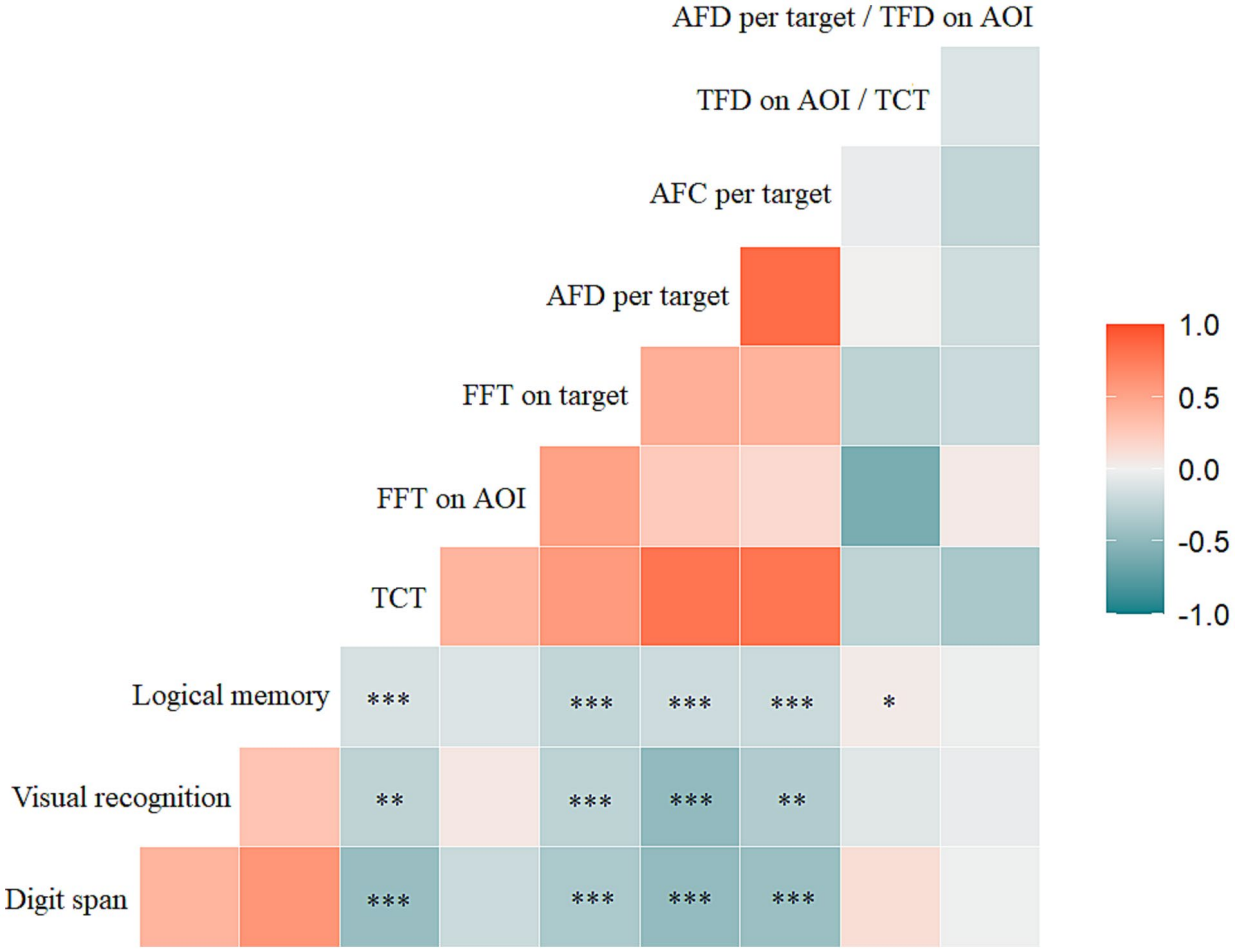
Correlations between the scores of memory scale assessment (digit span, visual recognition, logical memory) and TCT, FFT on AOI, FFT on target, AFD per target, AFC per target, TFD on AOI/TCT, AFD per target/TFD on AOI. Statistical significance for each task between groups is indicated by asterisk(s) (^*^p < 0.05, ^**^p < 0.01, ^***^p < 0.001). TCT, total completion time; FFT, first fixation time; AOI, area of interest; AFD, average fixation duration; AFC, average fixation count; TFD, total fixation duration.

The total completion time of the decoding phase was negatively correlated with the scores of the digit span (rs = −0.525, *p* = 0.000), visual recognition (rs = −0.315, *p* = 0.002), and logical memory (rs = −0.398, *p* = 0.000) (Figure 6).

First fixation time on targets in the decoding phase was negatively correlated with the scores of the digit span (rs = −0.406, *p* = 0.000), visual recognition (rs = −0.370, *p* = 0.000), and logical memory (rs = −0.374, *p* = 0.000). The average fixation duration per target in the decoding phase was negatively correlated with the digit span (rs = −0.511, *p* = 0.000), visual recognition (rs = −0.367, *p* = 0.000), and logical memory (rs = −0.378, *p* = 0.000) scores. The average fixation count per target was also negatively correlated with the scores of digit span (rs = −0.515, *p* = 0.000), visual recognition (rs = −0.291, *p* = 0.004), and logical memory (rs = −0.361, *p* = 0.000). There was a positive correlation between the total fixation duration on AOI/total completion time and logical memory score (rs = 0.262, *p* = 0.011) (Figure 6).

## 4 Discussion

In this study, we confirmed the presence of memory deficits among FLE patients in combination with scales and short-term memory tests by eye tracking technology. We found that FLE patients showed intact attentional alertness but impaired attentional retaining in memory processes. In addition, these differences in eye tracking metrics appeared mainly during the memory decoding phase and performed worse with both fractal and front-facing image stimuli. Poor performance in memory scales associated with the eye tracking data suggests that eye tracking technology has the potential to be used as a supplementary neuropsychological tool for memory evaluation.

It has been widely accepted that patients with FLE do possess memory impairments, showing dysfunction during encoding, free recall, and retrieval (Exner et al., 2002, Nolan et al., 2004), and there is also evidence of memory deficit following frontal lobe resection for epilepsy (McDonald et al., 2001). In our study, FLE patients performed worse on both the WMS-RC test and the eye tracking-based short-term memory tests, confirming the view that memory deficit is one of the long-term effects of FLE on the neural networks involved in memory function. The medial prefrontal cortex, along with an intact connection with the medial temporal lobe and hippocampus, has been involved in memory encoding and retrieval (Centeno et al., 2010; Squire et al., 2015; van den Berg et al., 2021). Petrides and Pandya (1988) have identified a larger group of fiber projections in the frontal cortex that originated from the various areas of the temporal region, which might be the anatomic basis of the memory circuit. The rich interconnectivity between the temporal and frontal lobes may facilitate epileptic activity propagation and subsequent dysfunction in distant structures. Additionally, a series of functional neuroimaging findings have revealed that the frontal lobe participated in supporting effective memory in conjunction with memory-associated structures like the hippocampus (Centeno et al., 2012; Braakman et al., 2013). Therefore, we inferred that the memory deficits in FLE might be partly due to the dysfunction of different regions within the frontal lobes contributing to long-term memory functioning (Patrikelis et al., 2009; Kibby et al., 2019; Caciagli et al., 2023).

The frontal lobe is the hub of the attentional cognitive control network, also involved in the orchestrating functions of attention. Thus, whether the memory impairment among patients with FLE was dependent on attention deficit was further investigated. We found no difference in performance between FLE and HC in the first fixation time on target in the memory encoding phase, which means the attentional alerting is mostly retained in FLE to guarantee that they are in a heightened sensitive state and have the ability to respond fast and correctly (Posner and Petersen, 1990). However, the following sustained attention during the retrieval period has been identified as obviously impaired due to a lower ratio of the total fixation duration on AOI/total completion time among patients with FLE. Previous studies have shown that the frontal cortex not only produces regulatory signals related to attention but also functions to maintain and control spatial attention (Fiebelkorn and Kastner, 2020). Thus, we inferred that the recurrent epileptic discharges and pathology in different frontal regions would interfere with the attentional network and further negatively affect higher cognitive functions such as attention and memory (Rayner et al., 2015; Fiebelkorn and Kastner, 2020).

We also noticed that the patients with FLE showed impairments primarily in recognition memory, which aligns with previous reports by Centeno et al. (2010). They observed that poor memory performance in FLE was caused by difficulties in information retrieving, rather than encoding dysfunction (Centeno et al., 2010), resulting in repeatedly searching for the correct target, corresponding to longer fixation duration and more fixation counts on the target captured. Why these difficulties in recalling memories mostly contributed to memory deficits in FLE patients is of interest. We believe a possible reason is that the damaged prefrontal cortex from FLE disturbs the memory decoding process. The hippocampus is responsible for the initial memory encoding, while the prefrontal cortex takes charge of memory retrieval and consolidation (Preston and Eichenbaum, 2013; Hainmueller and Bartos, 2020). During the memory recall phase, the prefrontal cortex receives visual input, integrates prior knowledge and short-term memory from the hippocampus, and ultimately regulates attentional allocation (Buschman and Miller, 2007; Katsuki and Constantinidis, 2014). Consequently, when the frontal lobe and affective cognitive network exhibit structural or functional abnormalities, memory recollection in the decoding phase suffers. Accumulating evidence has supported this hypothesis by showing that patients with right frontal lobe tumors performed poorly in the retrieval process (Anderson et al., 2011). Our study involved six patients diagnosed with FLE, specifically experiencing frontal lobe absence seizures. Drawing from previous research on absence seizures, we hypothesize that abnormal discharges originating in the frontal lobe contribute to cognitive impairment and disturbances in consciousness among these patients (Lenkov et al., 2013; Wang et al., 2023). In forthcoming research, the integration of eye movement techniques with advanced methods like functional magnetic resonance imaging (fMRI) and magnetoencephalography (MEG) could further explore the influence of the frontal lobe on absence seizures.

There is also a significant correlation between the total completion time of the memory game and the scores of the scales. Previous studies have found that the eye tracking index correlates well with cognitive performance. Therefore, we believe that eye tracking technology combined with cognitive tasks could be applied as an alternative option for neuropsychological assessment in clinical practice and long-term monitoring. Considering our finding of short-term memory deficit, probably due to attentional maintenance dysfunction during memory retrieval, future psychological training strategies, which focus on cognitive adjusting to sustain attentional focus, are suggested to cope with those problems and to improve memory.

## 5 Limitations

Nevertheless, there are several limitations in our current study. Firstly, it was a clinical study conducted at a single center with small sample size; prospective studies with larger participation with long-term follow-up are required. Secondly, lateralization of FLE would have an effect on cognitive performance. For better screening of the memory deficit profile of FLE, future studies should take lateralization into consideration. Moreover, the impact of anti-seizure medications on cognition is an inevitable confounding factor that limits our statistical power, newly diagnosed patients should be enrolled to exclude the interfering effects of drugs. Additionally, we can design new memory game paradigms that combine fractal pictures and frontal-facing images, which can facilitate further investigation of the connection and difference between general and social cognition.

## 6 Conclusion

Our study innovatively combined traditional scales and eye tracking short-term memory tasks, to explore the characteristics and mechanisms of working memory impairment in patients with FLE. We found that short-term memory deficits in FLE patients are probably due to attentional maintenance dysfunction during the memory retrieval phase. Eye tracking technology with cognitive tasks could be a reliable potential supplementary neuropsychological tool for cognition evaluation and precise process intervention.

## Data availability statement

The raw data supporting the conclusions of this article will be made available by the authors, without undue reservation.

## Ethics statement

The studies involving humans were approved by Ethics Committee of Xiangya Hospital of Central South University. The studies were conducted in accordance with the local legislation and institutional requirements. The participants provided their written informed consent to participate in this study.

## Author contributions

QZ: Data curation, Formal analysis, Investigation, Writing – original draft, Writing – review & editing. WS: Formal analysis, Methodology, Software, Visualization, Writing – review & editing. KH: Data curation, Writing – review & editing. LQ: Writing – review & editing. SW: Data curation, Writing – review & editing. XL: Writing – review & editing. QW: Software, Writing – review & editing. LF: Conceptualization, Project administration, Supervision, Writing – review & editing.

## Footnotes

^1^ http://mmlab.ie.cuhk.edu.hk/archive/facesketch.html
